# A Weekly Diary Study on Playful Study Design, Study Engagement, and Goal Attainment: The Role of Proactive Personality

**DOI:** 10.1007/s10902-022-00593-4

**Published:** 2022-11-03

**Authors:** Huatian Wang, Yue Ren, Wei Liu

**Affiliations:** 1 Department of Applied Psychology, Lingnan University, Hong Kong, SAR, China; 2 School of Economics, Qingdao University, Qingdao, Mainland China; 3 Faculty of Industrial Design Engineering, Technical University Delft, Delft, The Netherlands; 4 Department of Industrial Engineering and Innovation Sciences, Eindhoven University of Technology, Eindhoven, The Netherlands

**Keywords:** Playful study design, Study engagement, Goal attainment, Diary study, Proactive personality

## Abstract

Students’ learning processes are heavily impeded by the COVID-19 pandemic. Students are experiencing more online learning environment and less face-to-face idea exchange, which may make them feel exhausted and demotivated. Using self-determination and proactivity theories, we propose and examine whether playful study design (PSD)—a proactive study strategy including designing fun and designing competition in learning tasks—is effective in fostering study engagement, which, in turn, improves study goal attainment during the COVID-19 period. Moreover, we examine whether students who are high in proactive personality will benefit more (e.g., reach a higher level of study engagement) when using the PSD strategy. We collected data using a weekly diary approach during four consecutive weeks, including 97 people and 308 within-person observations. Results of multilevel analyses showed that weekly PSD was positively related to weekly study engagement, and in turn, facilitated weekly goal attainment. Moreover, we found that proactive personality moderated and strengthened the positive associations between PSD and goal attainment, study engagement and goal attainment, but not for the relationship between PSD and study engagement. Overall, we provide one of the first attempts to demonstrate how PSD strategy can be used in student study life to improve study engagement and reach their goals. We shed light on how proactive personality can safeguard the success of PSD strategy. Theoretical and practical contributions are discussed.

## Introduction

Study takes up a major part of campus life and the attainment of various study goals (e.g., meeting deadlines, taking exams) greatly improves student well-being and performance (Bakker et al., 2015; Fredricks et al., 2004; Pfeiffer & Pinquart, 2012). Research suggests that improving study engagement and attaining challenging study goals is vital to a student’s educational success and development into a competent member of society (Salmela-Aro & Upadyaya, 2014). Students who engage in, control, and attain their goals are more likely to show more energy, vigor, and enthusiasm to their learning tasks (Bakker et al., 2015; Seibert et al., 2001). From the educational intuitional perspective, previous studies provided important insights into what teachers and universities can do to improve student engagement and performance, such as the Self-Determination Learning Model of Instruction that can be used in the classroom to enhance student motivation for learning (Kim & Park, 2012; Wehmeyer et al., 2000), cognitive repertoire (Fredrickson, 2001), planned behavior (Abraham & Sheeran, 2003), library resources and services, students’ grants, and cultural activities (Salanova et al., 2010).

However, one of the stakeholders that have received little scholarly attention is the behaviors/strategies that students *themselves* can take to cope with various novel study challenges and tasks (Bakker & Van Woerkom, 2017). This is important because the self-initiated strategies tend to be more intrinsically motivating and reflect students’ own preferences, interests, and personal needs (Siu et al., 2014). We argue that besides the efforts of teachers and universities, students themselves can also proactively create better study environment and engage in effective and innovative study strategies, thereby maximizing their study motivation and seeking study resources (Robins et al., 2015; Wang et al., 2021). As such, proactive students can take appropriate actions in accord with their study goals, be energized to deal with study challenges and difficulties, and seize valuable opportunities and resources (Parker et al., 2010). More importantly, with the emergence of the COVID-19 pandemic, some educational institutions were forced to shut down and adopt alternative educating methods (e.g., online learning methods) (Maatuk et al., 2022). This prompts students to have more personal initiatives than ever before because they need to adjust their study strategies, develop new study methods, and complete study assignments in an innovative manner. Yet, we found that only a small handful of studies investigating the role of student-initiated strategies on study outcomes, such as self-regulation (Balkis & Duru, 2016), self-assessment (Andrade, 2019), and using personal resources (e.g., study self-efficacy, optimism, and hope) (Robins et al., 2015).

Therefore, building on limited previous work and the self-determination perspective (Ryan & Deci, 2000), in the current study, we propose a newly emerged concept—playful study design (PSD; Scharp et al., 2019) and examine whether this strategy has the potential to increase study engagement and goal attainment. The concept of playful design originated from the work setting where workers can proactively and purposively adjust the way they work by adding more fun and competition, so that they become more engaged in work tasks (Scharp et al., 2019). Inspired by this, we propose that the playful design can also be applied in the educational setting and be beneficial to students and their study process. Accordingly, we define PSD as a self-determined and proactive behavioral strategy that creates more fun and challenge onto the existing study tasks. Similar to the playful design in the work setting (Scharp et al., 2019), PSD includes two essential components: designing fun and designing competition. Thus, we argue that students may become more engaged when they change the way of conducting tasks by increasing fun and creating more competitive activities.

Following the self-determination and proactivity theories (Parker et al., 2010; Ryan & Deci, 2000), we aim to examine how PSD strategy can positively relate to study goal attainment via increased study engagement. Moreover, we argue that the extent to which students may benefit more from PSD strategy. In other words, whether students may differ in their personal traits/abilities to unlock the potential of PSD strategy. Following the proactive personality literature (Grant & Ashford, 2008), we examine whether proactive personality may moderate the effects of PSD on study engagement and goal attainment, such that students who are high in proactive personality can reach a higher level of study engagement when using PSD.

To thoroughly examine these propositions, we used a quantitative weekly diary approach (Ohly et al., 2010). Specifically, we collected data from university students on a weekly basis within four consecutive weeks (*N-between* = 97; *N-within* = 308). That said, we examine the dynamic fluctuations in PSD, study engagement, and study goal attainment within individuals. This enabled us to reveal how PSD acted as a more dynamic action influencing the changes of study engagement and goal attainment within a relatively short period of time (i.e., week), rather than seeing it as a one-time event. This within-person investigation is essential because students may vary in their actions, emotions, and tasks across days and weeks. The vast majority of studies on student performance have examined relatively stable differences between students in terms of their enduring engagement and behaviors (Haase et al., 2008; Salanova et al., 2010). Such between-person studies cannot explain why even highly engaged students may have an off-day and sometimes show below-average or poor performance (Bakker et al., 2015). In a recent literature review, Xanthopoulou and Bakker (2013) concluded that the average amount of variance in individual states (e.g., engagement) attributed to within-person (e.g., daily level) fluctuations is no less than 42%. Thus, employing a weekly diary design could provide insights into the dynamic fluctuations of students’ behaviors, states, and performance.

The current study makes three important contributions to the literature. First, we add to the study engagement and performance literature (Bakker et al., 2015) by suggesting a novel and specific behavioral strategy, namely playful study design. We shed light on *what* students can do to facilitate their study engagement and study goal attainment, especially during the COVID-19 period when students are required to take more personal initiatives to their study. Second, we attempted to identify the underlying mechanism (*how*) through which PSD may facilitate study goal attainment, and *when* students will benefit most from this self-determined behavioral strategy. We extend the proactivity literature (Parker et al., 2010, 2019) by uncovering student proactive study process and identifying the mediating role of study engagement and the moderating role of proactive personality. Third, using a weekly diary approach, we further reveal how students engage in PSD strategy on a weekly basis, and in turn, influences the weekly fluctuations in study engagement and goal attainment. Thus, we shed light on a more dynamic and ongoing process on the relationships between PSD, study engagement, and goal attainment within individuals.

## Theory and Hypotheses Development

### Self-determination Theory and Proactive Behaviors

Self-determined strategies mean “acting as the primary causal agent in one’s life and making choices and decisions regarding one’s quality of life free from undue external influence or interference.” (Wehmeyer, 1996, p. 22). On this notion, self-determined strategies enable a person to manage their goals, adjust to changing environments, and have stronger intrinsic motivation (Bakker & Van Woerkom, 2017). For example, Bakker and Van Woerkom (2017) propose that individuals can also use four proactive strategies (strengths use, playful design, job crafting, and self-leadership) to facilitate flow experience (a positive short-term state characterized by enjoyment, intrinsic motivation, and absorption). In a similar vein, Bakker (2017) indicate four bottom-up proactive and strategic approaches that may foster work engagement, including strengths use, self-management, job crafting, and mobilizing ego resources.

According to self-determination theory (SDT), one of the basic assumptions in the positive links between proactive behaviors and engagement is that proactive behaviors help to satisfy basic psychological needs for (either of) competence, autonomy, and relatedness (Ryan & Deci, 2000). For example, when individuals use playful work/study design, they can establish a better balance between their skills and task challenges, satisfying their needs for competence. Also, individuals may reach a closer relationship with their classmates/colleagues when using humor and wit in a conversation, which helps meet their needs for relatedness. The satisfaction on basic needs, in turn, increases intrinsic motivation because students feel more fulfilled (Ryan & Deci, 2000), enabling them to have more energy and vigor for conducted tasks, which may in turn facilitate goal attainment. Therefore, the self-determined strategies can make individuals produce stronger engaged states.

Furthermore, the scholars in SDT suggest that not all individuals benefit from the self-determined strategies equally and that personal traits/personalities should be taken into consideration to explain the personal variations (Crant, 2000; Parker et al., 2010). Studies found that proactive-oriented personality is one of the personal factors for individual creativity (Gong et al., 2012), performance (Bakker et al., 2012), and career success (Seibert et al., 1999). Those with high proactive personality are more able to effectively mobilize their energy and emotions to deal with difficulties and challenges (Gong et al., 2012). Therefore, based on the SDT theory, in the following sections, we will develop hypotheses on how PSD (proactive study strategy) influences students’ study engagement, and in turn, enhances study goal attainment. Moreover, how proactive personality may play a moderating role among these relationships.

### Playful Study Design

PSD emerges from the interaction between self-determination and play literature (Bakker & Van Woerkom, 2017; Petelczyc et al., 2018). This concept has been investigated in the work domain (Bakker et al., 2020a, 2020b; Scharp et al., 2019, 2021), which is associated with higher work performance and well-being, but has seldom been applied in the study domain. However, according to Bakker et al., (2020a, 2020b, p. 3): “Study and sport/exercise are also structured, goal-directed, time demanding, and to a large extent obligatory, and both domains may be excellent areas for playful design.” PSD is comparable to other forms of proactive behaviors (e.g., job crating, voice, taking charge, etc., Parker et al., 2019), but fundamentally distinct from the latter as it emphasizes adding play elements to *existing* tasks, aiming to change task experience but without changing the task itself (Bakker et al., 2020a, 2020b). PSD is primarily developed based on two distinct clusters of play elements. One is ludic play, characterized by humor, excitement, and entertainment, which relates to one’s pleasure and enjoyment (Barnett, 2007). The other cluster is agonistic play, which is concerned with efforts, goals, and purpose, serving to pose challenges and helping a person grow and achieve a sense of meaningfulness (Caillois, 2001).

Based on the SDT and proactivity theories (Bakker & Van Woerkom, 2017; Crant, 2000), the manifestation of PSD implies that students take their own initiative to challenge the status quo by making their study more interesting and motivating, increasing the challenge when they feel study tasks boring. According to Scharp et al. (2019), students can either use designing competence or designing fun in PSD. For instance, when a student competes with him/herself to try to finish the tasks more quickly than before, this refers to designing competition; when a student uses humor or wit trying to make a meeting more interesting, this refers to designing fun. Specifically, on a weekly basis, perhaps certain week is not motivating, for example, students have to attend a course that they are not fond of than other weeks (Bakker et al., 2015). In this situation, PSD may be a good strategy to improve the excitement and involvement in that week. Moreover, during the COVID-19 period, students are probably experiencing more online courses and online assignments. PSD can be particularly useful here because it facilitates productivity, such as adding more online fun interactions with their tutors, asking an interesting question, or running a race with classmates to see who can finish the homework first.

### Study Goal Attainment

Allport (1947) argues that longer-term goals are important as they direct and make sense of everyday action. Goals are “internally desired states where states are broadly construed as outcomes, events or processes.” (Austin & Vancouver, 1996, p. 338). On this notion, goal attainment is essential for students as it relates to the sense of meaningfulness, self-actualization, and performance (Wehmeyer et al., 2003). Previous studies have highlighted the role of self-regulation (e.g., planned behavior) in the process of goal pursuit (Abraham & Sheeran, 2003; Sheeran et al., 2005). However, several other behavioral and intentional aspects are also noted by Abraham and Sheeran (2003) in the pursuit of goals, such as constructing behavioral selection, assessing the readiness of planned behavior, resolving the conflict between various goals. Similarly, Bagozzi (1992) noted that undertaking preparatory or “instrumental” actions are significant for individuals to actualize goals. These argumentations indicate that concrete behavioral strategy may matter when pursuing one’s goal. Particularly, due to the spread of the COVID-19, study processes (e.g., face-to-face communication, group discussion and assignment, and exams) are negatively hindered, which sets more challenges and difficulties in study goal attainment (Maatuk et al., 2022). A self-determination strategy for learning that can fit this unfavorable circumstance is urgently needed at this moment.

Based on the notion of PSD, learning tasks become more challenging and interesting when students use PSD, which likely increases students’ willingness and intrinsic motivation to conduct the tasks. The stronger motivation may, in turn, helps individuals to realize their goals because students are more energetic and enthusiastic towards the current task (Bakker et al., 2015; Wehmeyer, 1996). In addition, as the tasks become more fun and playful through PSD, students can enjoy study to a greater extent (e.g., produce a happier mood), which may enhance behavioral and cognitive repertoire in solving problems and stimulating goal attainment (Fredrickson, 2001). Thus, as the proactivity theory suggests, PSD may be linked to goal attainment as it changes the perception, motivation, and strategy to perform tasks. Indeed, several studies have indicated the potential links between PSD and goal attainment in the work context. For example, Scharp et al., (2019, 2021) have shown that playful design indirectly associates with employees’ creativity through engagement. Liu et al. (2021) showed that PSD is positively related to workers’ flow experience and less fatigue, which in turn, fosters work performance. Bakker et al., (2020a, 2020b) have found that naval cadets have higher performance on the days when they use playful design. Thus, combining self-determination theory and proactivity theory, we argue that PSD will be positively related to goal attainment in the study context.

#### H1a

Designing fun is positively related to study goal attainment.

#### H1b

Designing competition is positively related to study goal attainment.

### The Mediating Role of Study Engagement

Study engagement is a complex concept that may comprise behavioral, emotional, and cognitive aspects of engagement (Fredricks et al., 2004). In the current study, we define study engagement based upon Schaufeli et al. (2002)’s definition of engagement. He refers to engagement as a positive and fulfilling state of mind which is characterized by vigor, dedication, and absorption. To be specific, “vigor refers to high levels of energy and resilience while studying. Dedication is characterized by being strongly involved in one’s studies and experiencing a sense of significance and enthusiasm. Absorption is the state of being fully concentrated and engrossed in one’s study activities” (Bakker et al., 2015, p. 50).

PSD may indirectly facilitate goal attainment through study engagement. There are several reasons for this. First, designing fun and competition may establish a better balance between one’s skills and task challenges and achieve a better person-environment fit. Research shows that skill-challenge balance is crucial for autotelic and optimal experience (e.g., absorption and engagement; Csikszentmihalhi, 2020). Second, as the SDT theory suggests, students would become more intrinsically motivated as the study tasks are not as boring as before. For example, Siu et al. (2014) have tested intrinsic motivation as a mediator between personal resources and study engagement, confirming its mediating role. Third, according to self-determination model of flow (Bakker & Van Woerkom, 2017), PSD may help satisfy basic psychological needs such as competence, autonomy, and relatedness, which in turn fosters flow (a concept which is theoretically linked to engagement). For example, when a student tries to make conversation more interesting and fun using humor and wit, they may establish a closer connection with classmates as they enjoy the conversation to a greater extent (relatedness). Empirical studies have shown that engagement may be potentially fostered when individuals/students use proactive behavioral strategies (Bakker et al., 2015; Liu et al., 2021; Pfeiffer & Pinquart, 2012).

When students are engaged in the tasks at hand, they tend to have more energy dedicated to the performed tasks (Schaufeli et al., 2002). Engaged students tend to be intrinsically devoted to investing in learning, attending classes, and participating in study activities as they likely enact more energetic behavior and exert more efforts. Students divert and mobilize their cognitive resources to the targeted tasks, attempting to solve the encountered problems until they are solved because students perform the tasks because of personal interests rather than extrinsic rewards (Liu et al., 2021).

Prior some studies assessed the quantitative associations between adolescents’ goal engagement and the attainment of various goals. For example, Berzonsky and Kuk (2000) found that goal engagement is positively associated with achieving developmental goals. Haase et al. (2008) showed that a higher level of goal engagement predicts career success. Also, Kim and Park (2012) have shown that engagement (experimental group, 12 students with their families and special educators) considerably improves goal attainment than the control group (12 students no treatment). With 133 adolescents with visual impairment and in 449 sighted peers, goal engagement predicts stronger progress in goal attainment with respect to career choice and romantic relationships (Pfeiffer & Pinquart, 2012). Bakker et al. (2015) have found that study engagement fully mediates the effects of personal resources on study performance. Based on the literature of proactivity and engagement, we hypothesize:

#### H2

Study engagement mediates the relationship between playful study design (a: designing fun; b: designing competition) and study goal attainment.

### The Moderating Role of Proactive Personality

Proactivity, in general, refers to behavioral or cognitive aspects that individuals take their own initiative and anticipatory actions to plan and change themselves or the environment in a desirable direction (Grant & Ashford, 2008; Seibert et al., 2001). Accordingly, proactive students might conduct their learning tasks in a more self-anticipatory, self-initiated, and active manner to satisfy their basic needs and meet their goals. As aforementioned, PSD may indirectly facilitate goal attainment through study engagement, but we propose that the effects may depend on proactive personality (PP) because proactive students are more willing and prepared to challenge the status quo, change themselves to adapt to learning environments, and improve study challenges to an optimal level (Elsaied, 2019; Fuller Jr & Marler, 2009).

Proactive individuals are better able to acquire social resources, prevent potential loss, and keep their performance. When using PSD, students who are high in PP may benefit most from the activities as they tend to be more immersed and enjoy the learning tasks more intensely. When students try to improve task challenges, proactive students can reach an optimal level of task challenge as they are more sensitive to cues in the environment and keep challenging the status quo until they are satisfied with the current situation (Bakker & Van Woerkom, 2017). Similarly, when they are engaged to the task at hand, they may direct and mobilize their cognitive and motivational resources towards the learning targets, which enables them to be more likely to achieve their goals. For example, as Elsaied (2019, p. 228) noted: “highly proactive persons identifying opportunities and taking action on them, showing initiative and preserving until they succeed, tend to be keen on harnessing all resources available to achieve their goals and objectives.”

At the same time, from a goal-setting perspective (Seibert et al., 1999), proactive individuals have broader behavioral and cognitive repertoires and are more innovative (e.g., political knowledge, voice, career initiative). This broadened repertoire indicates that they have greater opportunities to achieve goals because they can have access to more social and intellectual resources. The self-determination model of flow (Bakker & Van Woerkom, 2017) demonstrates that key resources, including emotional intelligence and proactive personality, may potentially moderate the effects of proactive strategies on flow experience (a theoretically relevant concept to engagement but focuses more on short-term state). Indeed, PP has been shown to heighten performance and acquire more rewards (Seibert et al., 2001). Several studies have reflected that individuals who have high PP are more likely to achieve career success, better performance, and satisfaction with their lives (Bakker et al., 2020a, 2020b; Liu et al., 2021; Scharp et al., 2019; Seibert et al., 2001). Allen et al. (2006) have shown that individuals who have high PP are more motivated to enact changes, actively solve problems, and pursue opportunities that enable the advancement of their interests and careers. In a similar vein, Li et al. (2014) found that PP moderates the effects of social support on work engagement. Finally, a recent article of Yi-Feng Chen et al. (2021) showed that during the COVID-19 period, doctors and nurses with higher PP were associated with higher levels of performance and well-being when their work routines were disrupted. Thus, PP was an important dispositional resource that can help individuals deal with difficult and uncertain situations more effectively. Likewise, we believe that students with higher PP are able to design their study tasks and environment in a more meaningful and creative way, and reach a higher level of study engagement even during the COVID-19 period. Correspondingly, based on the proactivity theory, we hypothesize that:

#### H3a

Proactive personality moderates the relationship between playful study design and study engagement, such that this relationship is stronger when students score higher (vs. lower) in proactive personality.

#### H3b

Proactive personality moderates the relationship between playful study design and study goal attainment, such that this relationship is stronger when students score higher (vs. lower) in proactive personality.

#### H3c

Proactive personality moderates the relationship between study engagement and study goal attainment, such that this relationship is stronger when students score higher (vs. lower) in proactive personality.

## Method

### Procedure and Participants

To test our hypotheses, we conducted a weekly diary study. A diary study design aims to capture individual states, moods, behaviors, and performance within a short period of time with repeated measures (Ohly et al., 2010). Many scholars have realized the methodological advantages of using a diary design because it captures “life as it is lived” (Bolger & Laurenceau, 2013; Ohly et al., 2010). A diary study has its merits given that one can have different levels of states, behaviors, and/or performance in different days and weeks based on various dynamic factors and contexts (Gabriel et al., 2019). Using a diary study, we can effectively control and examine the effects of previous states and behaviors on the current period’s states and performance (Demerouti & Cropanzano, 2017), which cannot be revealed in a cross-sectional design. Research also shows that the diary design can reduce possible retrospective bias and increase the likelihood of causality for the studied relationships since it includes repeated measures (Gabriel et al., 2019; Ohly et al., 2010). Thus, in the current study, using a weekly diary design, we did not focus on the traditional paradigm of how those engaging in PSD (*vs.* those not) increases study engagement and study goal attainment (i.e., between-person), but we expect to shed light on how playful study design strategy associates with study outcomes over time (i.e., within-person).

We conducted a weekly diary study using a sample of 1-year college students at a business school in Qingdao, China. The questionnaire was distributed via virtue links. A weekly diary design included two parts: first, participants were required to fill in a general questionnaire where we welcomed and explained our study goal. To reduce common method bias, we only presented participants that this study aimed to record their weekly study process and performance. The confidentiality and anonymity of responses were assured. Also, in the general questionnaire, we asked students to fill in their demographic information including age and gender and rate their proactive personality. Besides, we asked students to create a unique identification code, so that we could use it to match the following weekly responses from each student. Subsequently, we sent the weekly questionnaire links every Friday to students’ WeChat account (an app for instant message in mainland China), so that the students can reflect upon their feelings, experiences, and behaviors for the current week. That said, in each week, students can freely rate their weekly study process including PSD, study engagement, and study goal attainment. The contact list was based on the network of the second author. The second author was responsible for delivering the questionnaires, including sending them to students’ WeChat account, reminding deadlines, and resolving any potential technical problems. One administrative assistant was also involved in assisting the second author to deliver the questionnaire link. The questionnaire link was valid until the end of Sunday, so they were unable to complete the questionnaire after Sunday. Also, we kindly asked students to complete the questionnaire as soon as possible since the questionnaire would take less than 10 min. To reduce the common method bias, as suggested, we did not interact and communicate with students, so that we decreased our potential impact on their self-evaluation. We also control for the order effects of items in each diary (Wang et al., 2021). In conclusion, we collected data 4 times within four weeks. That is, the general questionnaire and week one questionnaire were at T1; the following three weekly questionnaires were at T2, T3, and T4.

Before recruiting participants, we did a power analysis to determine the (minimum) sample size. Results showed that at least 36 individuals with four-time repeated measures were required if statistical power was expected to be above 95%. In organizational studies, researchers suggest that, on average, 83 individuals participating in a diary study would be sufficient to meet the statistical power (Gabriel et al., 2019). Conclusively, we decided to recruit 90 participants. During the data collection stage, 97 participants originally filled in the general question. However, during the weekly questionnaires, we lost some participants. 97, 85, 83, and 91 participants responded to the weekly questionnaire at T1, T2, T3, and T4, respectively. Following the multilevel (longitudinal) study practices, we excluded those participants who filled in weekly questionnaires only once. Finally, we matched the multilevel data with 77 participants filling in weekly questionnaires for four times. We yielded 308 data observations (i.e., 77 participants × 4 weeks; response rate 79.38%). Students’ average age was 19 (*SD* = 0.81). 36.1% of them were male.

### Measurement

We administered the questionnaires in Chinese and followed the back-translation procedure to ensure consistency (Brislin, 1976). First, we translated the items into Chinese. Then we asked another professional linguist (an English teacher) to perform a literal translation of the items back to English. Finally, we compared the back translation to the original text to make sure the back translation is accurate and complete. We adjusted the items to fit the weekly context.

*Weekly playful study design* was measured using the scale developed by (Scharp et al., 2019). We identified two aspects of playful study design: *designing fun* and *designing competition*. We adjusted the items to fit the school context. Among them, four items were used to measure designing fun. An example item was like “This week, I approached my study tasks creatively to make them more interesting.” Cronbach’s α = 0.93, 0.96, 0.95, 0.95 at T1, T2, T3, T4. Five items were used to measure designing competition. An example item was like “This week, I approached my study tasks as a series of exciting challenges.” Cronbach’s α = 0.91, 0.96, 0.94, 0.92 at T1, T2, T3, T4. Items were rated using a 5-point Likert scale ranging from 1 (*never*) to 5 (*very often*).

*Weekly study engagement* was measured using the scale developed by (Schaufeli et al., 2002), including the dimensions of energy (e.g., this week, when I studied, I felt that I was bursting with energy; 2 items), dedication (e.g., this week, I was enthusiastic about my studies; 2 items), and absorption (e.g., this week, time flew when I was studying; 2 items). Responses were rated on a 7-point scale ranging from 1 (*never*) to 7 (*always*). Cronbach’s α = 0.95, 0.96, 0.97, 0.97 at T1, T2, T3, T4.

*Weekly study goal attainment* was self-evaluated by students from 0% (no attainment) to 100% (complete attainment) for each week’s study tasks (see, Grant et al., 2009). We transformed it into a 10 scale (i.e., 0 for no attainment, 10 for complete attainment) to align with the scaling of other measurements. For the single item, we further used the test–retest approach to examine its reliability (Shen et al., 2019). We compared T1 with T2, T3, and T4, respectively, to calculate the intraclass correlation. We found that the intraclass correlation was 0.67 between T1 and T2; 0.79 between T1 and T3; 0.72 between T1 and T4. All the values were acceptable.

*Proactive personality* was measured by the scale developed by Bateman and Crant, (1993). A 7-point Likert scale was used, ranging from 1 = *strongly disagree* to 7 = *strongly agree*. We only measured it once in the general questionnaire at T1. An example item was like “If I see something I don’t like, I fix it.” Cronbach’s α = 0.91 (5 items in total).

Control variables. We included age and gender as controls because these demographic variables may influence student engagement and performance (Bakker et al., 2015). Besides, research also suggests that study goal attainment may be influenced by the extent of difficulty the goal is and how much time one spends in achieving goals (Grant et al., 2009). Thus, we also considered *goal difficulty* and *goal time spending* as controls. Specifically, goal difficulty was measured by an item “This week, how difficult you felt when you pursued the completion of study goal or task?”, ranging from 1 = *very easy* to 4 = *very difficult* (Grant et al., 2009). Goal time spending was measured from 1 = less than 1 h; 2 = 1 to 2 h; 3 = 2 to 3 h; 4 = 3 to 4 h; 5 = 4 to 5 h; 6 = more than 5 h.

Lastly, to improve the causality likelihood and overcome potential endogeneity, in line with suggestions (Bolger & Laurenceau, 2013), we also controlled for the lagged term (i.e., t−1 period) of the dependent variables and independent variables (i.e., the t−1 period of playful study design and t−1 period of study goal attainment).

### Statistical Approach

We used multilevel regression analysis by MLwiN software to analyze the diary data. The steps were as follows: first, we entered intercept and control variables (including age, gender, goal difficulty, goal time spending, and the lagged term of PSD and study goal attainment, see Model 0 in Table 2). Second, we entered the predictors (i.e., designing fun and designing competition) separately (see Model 1 & 3 in Table 2). Third, we entered the interaction terms between predictors and the moderator (i.e., proactive personality). See Model 2 & 4 in Table 2. Likewise, we used the same procedure to examine the moderating effect of proactive personality on the relationship between playful study design and study engagement; as well as the relationship between study engagement and study goal attainment, respectively (see Table 3). Specifically, we first entered intercept, control variables, and playful study design strategies, (see Model 1 & 3, Table 3). Subsequently, we entered the interaction terms between playful study design strategies and proactive personality (see Tables 2, 3, 4). These yielded the results of the moderating effect of proactive personality on the relationship between playful study design and study engagement (i.e., the first path). Finally, we entered the study engagement (Model 5, Table 3), and the interaction term between study engagement and proactive personality (Model 6, Table 3), respectively, in order to obtain the moderating effect of proactive personality on the relationship between study engagement and study goal attainment (i.e., the second path). When conducting the multilevel analyses, we examined the random effects of slopes and tested the improvement of each model compared to the previous model by computing the differences of log-likelihood statistic—2*log and subjected this difference to a χ^2^ significance test. All week-level variables were person-mean centered to avoid multicollinearity and spurious regression, as well as enabling us to solely focus on within-person relationships between the study constructs.

To examine the (multilevel) mediation effects, we used MLmed Macro (Rockwood & Hayes, 2017). This procedure can calculate 95% Monte Carlo confidence intervals based on 10,000 bootstrapping. As our data were nested in two levels, MLmed Macro is an appropriate tool to conduct such analyses, compared to the traditional PROCESS Macro, which cannot examine multilevel data.

## Results

### Preliminary Analysis

Table 1 showed means, standard deviations, and correlations among all the study variables. Since we had a multilevel data structure, we reported not only variables’ correlations on the between-person level but also correlations on the within-person level.

**Table 1:** Means, SD, within-person (below diagonal) and between-person (above diagonal) correlations among studied variables

		Means	SD	1	2	3	4	5	6	7
1	Playful study design (designing fun)	3.83	1.01		0.81**	0.68**	0.51**	– 0.48**	0.01	– 0.03
2	Playful study design (designing competition)	3.93	0.89	0.50**		0.76**	0.55**	– 0.53**	–0.04	0.03
3	Study engagement	5.41	1.33	0.44**	0.47**		0.54**	– 0.53**	–0.05	0.01
4	Study goal attainment	7.19	1.81	0.20**	0.22**	0.20**		– 0.38**	0.02	0.05
5	Proactive personality	2.55	1.15	0.02	0.01	–0.01	0.02		0.04	– 0.03
6	Age	18.76	0.81	0.01	–0.01	–0.02	0.02	0.04		– 0.16**
7	Gender	1.64	0.48	0.01	0.03	0.03	0.01	– 0.03	–0.16**	

*N* = 97 participants; *N* = 308 data points

^***^p < .001; **p < .01; *p < .05

We computed the ICC (intra-class correlation) to examine whether there was significant variance on the weekly level (i.e., within-person variance). Results showed that playful study design, study engagement, study goal attainment accounted for the variance of 54.63%, 59.22%, and 38.55%, respectively. These results supported the application of multilevel analysis and implied that students not only scored differently on the investigated variables but also showed variances over time within each student.

We also ran multilevel confirmatory factor analysis (MCFA) to explore the factorial structures of our measures using Mplus software. The analysis type was TWO LEVEL. Results showed that four distinguished latent constructs (i.e., playful study design as two factors—designing fun and designing competition, study engagement as one factor, proactive personality as one factor) had an acceptable model fit: χ^2^ = 646.489; df = 305; CFI = 0.92; TLI = 0.90; SRMR-_within_ = 0.06; SRMR-_between_ = 0.05; RMSEA = 0.05. This measurement model was significantly better than the model collapsing playful study design into one factor (χ^2^ = 833.604; df = 312; CFI = 0.87; TLI = 0.85; SRMR-_within_ = 0.08; SRMR-_between_ = 0.06; RMSEA = 0.07; Δχ2 (7) = 187.115, *p* < 0.001). To conclude, the multilevel CFA results supported the discriminant validity.

### Hypothesis Testing

Table 2 showed that weekly designing fun was positively related to weekly study goal attainment (*B* = 0.92, *p* < 0.001). Thus, Hypothesis 1a was supported. Weekly designing competition was positively related to weekly study goal attainment (*B* = 1.02, *p* < 0.001). Therefore, Hypothesis 1b was supported as well. This implies that playful study design, including designing fun and designing competition, can facilitate students’ study goal attainment on a weekly basis (Table 3).

**Table 2:** Multilevel regression results of playful study design and proactive personality predicting study goal attainment

	Model 0		Model 1		Model 2		Model 3		Model 4	
	*B*	*SE*	*B*	*SE*	*B*	*SE*	*B*	*SE*	*B*	*SE*
Constant	7.33***	0.34	7.21***	0.29	7.21***	0.30	7.26***	0.29	7.25***	0.29
Age	0.16	0.11	0.18	0.10	0.16	0.10	0.18	0.10	0.16	0.10
Gender	– 0.08	0.21	– 0.04	0.17	– 0.01	0.18	– 0.05	0.17	– 0.01	0.18
Goal difficulty	– 0.06	0.15	– 0.15	0.12	– 0.19	0.12	– 0.12	0.12	– 0.17	0.12
Goal time spending	0.47***	0.07	0.28***	0.06	0.29***	0.06	0.26***	0.06	0.27***	0.06
Designing fun (lag)			– 0.14	0.11	– 0.09	0.11				
Designing competition (lag)							–0.28*	0.13	– 0.19	0.13
Study goal attainment (lag)	0.40***	0.06	0.31***	0.06	0.29***	0.06	0.39***	0.06	0.35***	0.06
Designing fun			0.92***	0.10	0.83***	0.15				
Designing competition							1.02***	0.10	0.89***	0.16
Proactive personality					– 0.16	0.10			–0.15	0.10
Designing fun × PP					0.18*	0.07				
Designing competition × PP									0.19*	0.08
Level 2 variance	0.01	0.01	0.01	0.01	0.11	0.09	0.01	0.01	0.14	0.10
Level 1 variance	2.23	0.21	1.61	0.15	1.49	0.14	1.59	0.15	1.47	0.14
–2loglikelihood	847.99		770.88		761.92		770.17		761.34	
d.f			2		4		2		4	
Δ –2log likelihood			77.11***		86.07***		77.82***		86.65***	

*N* = 97 participants; *N* = 308 data observations; PP = proactive personality

^***^p < .001; **p < .01; *p < .05; "lag" refers to the lagged term

Level 2 variance refers to unexplained variation at the person level; level 1 variance refers to unexplained variation at the weekly level

**Table 3:** Multilevel regression results for the moderating effect of proactive personality (the first-path and the second-path)

	Study engagement								Study goal attainment			
	Model 1		Model 2		Model 3		Model 4		Model 5		Model 6	
	*B*	*SE*	*B*	*SE*	*B*	*SE*	*B*	*SE*	*B*	*SE*	*B*	*SE*
Constant	5.43***	0.21	5.48***	0.19	5.56***	0.18	5.59***	0.17	7.16***	0.29	7.08***	0.29
Age	0.02	0.07	– 0.01	0.06	0.05	0.06	0.03	0.06	0.20*	0.10	0.18	0.10
Gender	– 0.04	0.12	– 0.08	0.11	– 0.09	0.11	– 0.12	0.09	0.02	0.17	0.09	0.17
Goal difficulty	0.09	0.08	0.11	0.08	0.06	0.07	0.07	0.07	– 0.18	0.12	– 0.23	0.12
Goal time spending	0.09*	0.04	0.12**	0.04	0.07*	0.03	0.09*	0.04	0.26***	0.06	0.26***	0.06
Designing fun (lag)	– 0.02	0.09	– 0.02	0.09								
Designing competition (lag)					– 0.07	0.10	– 0.04	0.10				
Study goal attainment (lag)									0.34***	0.06	0.32***	0.06
Study engagement (lag)	0.11	0.07	0.04	0.07	0.13	0.07	0.07	0.07	– 0.17	0.09	– 0.15	0.09
Designing fun	0.96***	0.06	0.77***	0.07								
Designing competition					1.15***	0.06	0.98	0.07				
Study engagement									0.73***	0.07	0.67***	0.11
Proactive personality			– 0.35**	0.06			– 0.24**	0.06			– 0.12	0.10
Designing fun × PP			0.02	0.04								
Designing competitiom × PP							0.03	0.04				
Study engagement × PP											0.11*	0.05
Level 2 variance	0.07	0.04	0.07	0.04	0.06	0.04	0.06	0.03	0.01	0.01	0.08	0.06
Level 1 variance	0.62	0.06	0.54	0.05	0.49	0.05	0.45	0.04	1.57	0.15	1.45	0.14
–2loglikelihood	565.36		534.21		510.56		490.74		766.99		759.68	
d.f			2				2		2		4	
Δ –2loglikelihood			31.15***				19.82***		81***		88.31***	

*N* = 97 participants; *N* = 308 data observations. PP = proactive personality

^***^p < . 001; **p < .01; *p < .05; "lag" refers to the lagged term

Level 2 variance refers to unexplained variation at the person level; level 1 variance refers to unexplained variation at the weekly level

Table 4 revealed the mediation effect of study engagement. We found that, on the within-person level, designing fun was positively related to study goal attainment via increasing study engagement (Estimate = 0.34, 95% CI = [0.19, 0.52]), and that designing competition positively influenced study goal attainment via increasing study engagement (Estimate = 0.38, 95% CI = [0.14, 0.63]). Notably, we also reported the mediation effect of study engagement on the between-person level. Results showed that study engagement also mediated the relationship between playful study design and study goal attainment (Estimate = 0.76, 95% CI = [0.40, 1.12] for designing fun; Estimate = 0.75, 95% CI = [0.22, 1.30] for designing competition). Therefore, Hypothesis 2 was supported.

**Table 4:** Mlmed results for the mediation effects of study engagement

*Control for age, gender, goal difficulty, and goal time spending*	Estimate	*SE*	z-statistics	*p*	LL	UL
*Within-person leve:*						
Weekly designing fun weekly study engagement weekly study goal attainment	0.34	0.08	4.09	0.00	**0.19**	**0.52**
Weekly designing competition weekly study engagement weekly study goal attainment	0.38	0.12	3.10	0.00	**0.14**	**0.63**
*Between-person level*						
Weekly designing fun weekly study engagement weekly study goal attainment	0.76	0.18	4.12	0.00	**0.40**	**1.12**
Weekly designing competition weekly study engagement weekly study goal attainment	0.75	0.27	2.76	0.01	**0.22**	**1.30**

*N* = 97 participants and *N* = 308 data points. All the variables are person-mean centered; bootstrapping = 10,000; confidence level is at 95%

Bold indicates the significance

**Fig. 1:**
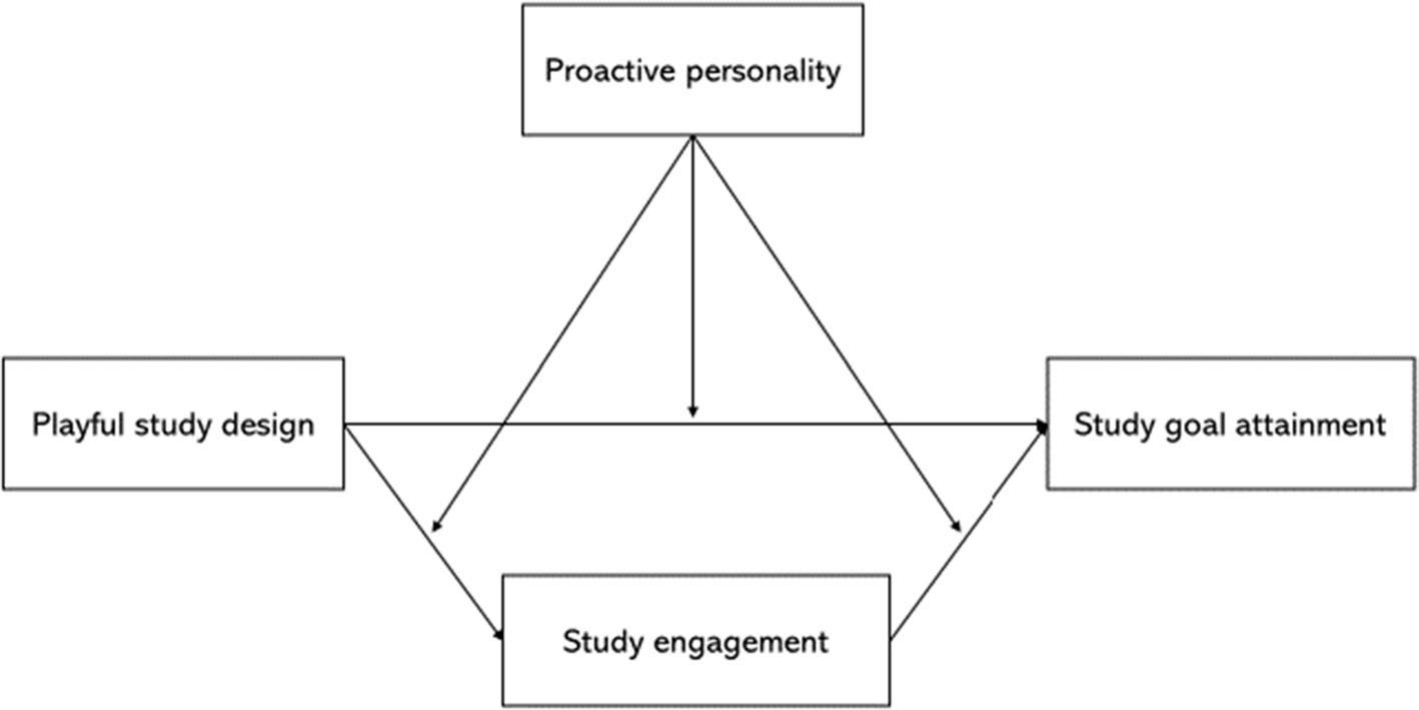
Conceptual model

**Fig. 2:**
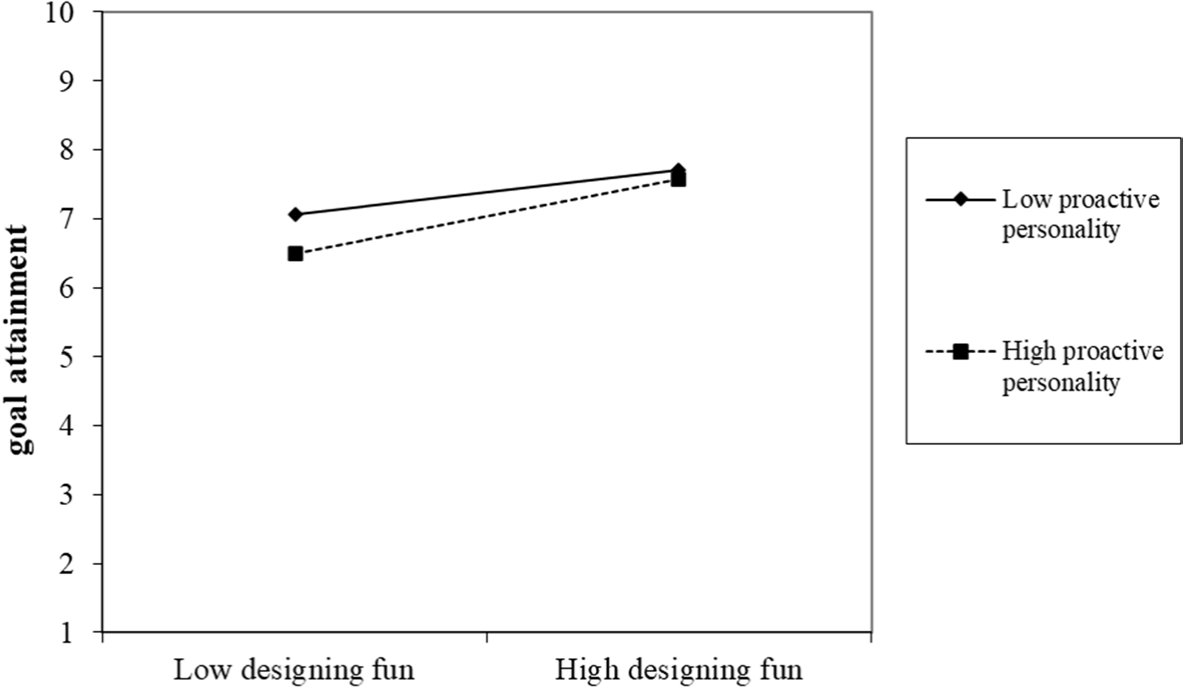
Two-way interaction terms between designing fun and proactive personality on study goal attainment

**Fig. 3:**
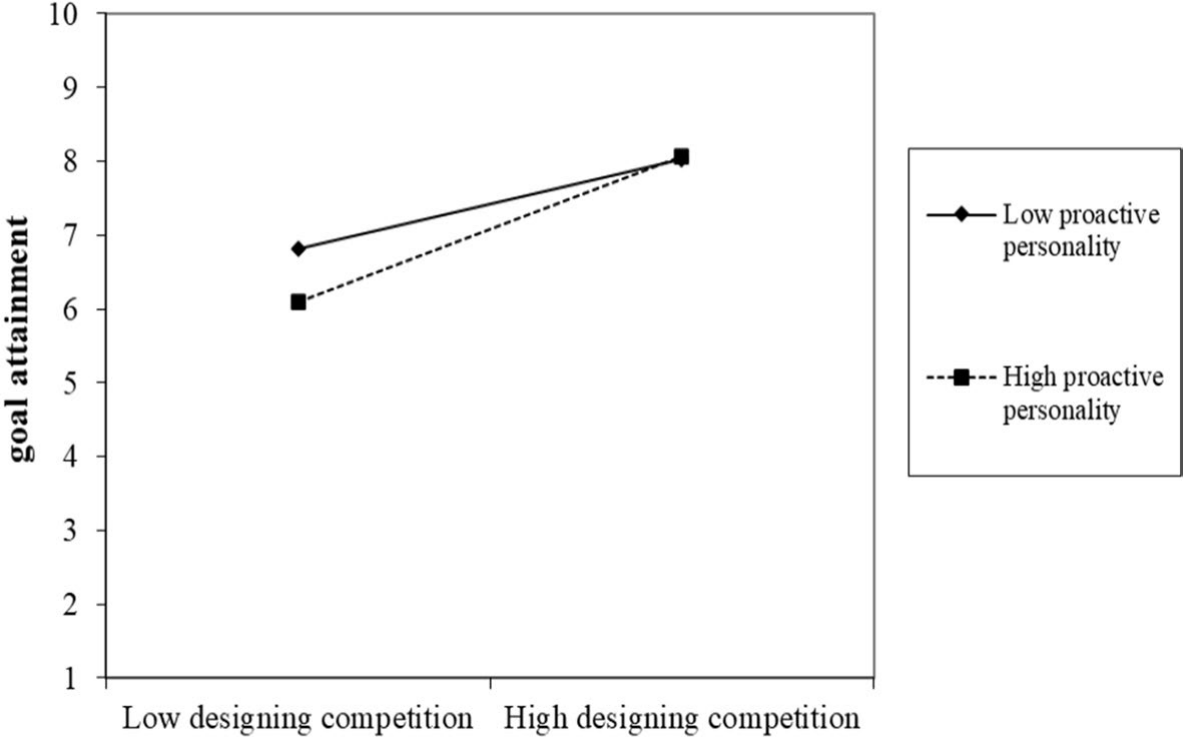
Two-way interaction terms between designing competition and proactive personality on study goal attainment

**Fig. 4:**
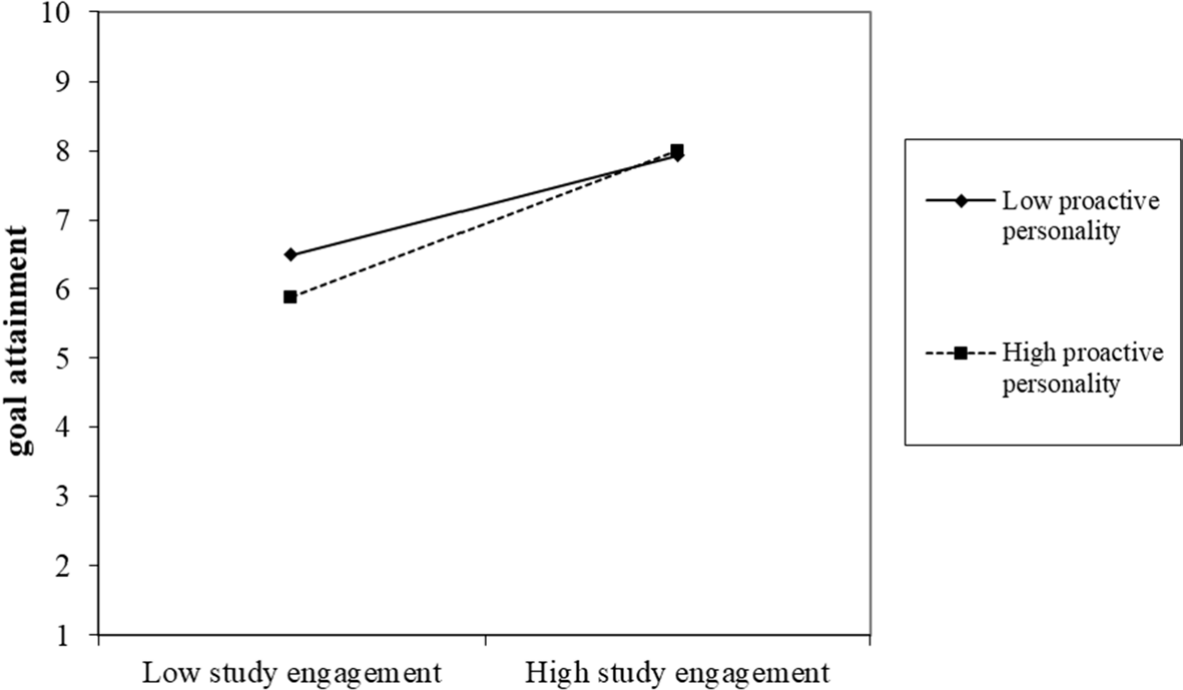
Two-way interaction terms between study engagement and proactive personality on study goal attainment

Hypothesis 3 indicates that proactive personality moderates the relationships between PSD, study engagement, and study goal attainment (Figs. 1, 2, 3, 4). First, we did not find the significant two-way interaction between playful study design and proactive personality on study engagement (*B* = 0.02, *p* = 0.62 for designing fun; *B* = 0.03, *p* = 0.45 for designing competition) (see Table 3, Model 2 and 4). Thus, Hypothesis 3a was not supported. Second, we found the two-way interaction between playful study design and proactive personality on study goal attainment was significant (*B* = 0.18, *p* = 0.01 for designing fun; *B* = 0.19, *p* = 0.02 for designing competition) (see Table 2). We further conducted simple slope test. Results showed that designing fun strategy was more positively related to study goal attainment when students have higher proactive personality (*B* = 1.04, *p* < 0.001) compared to when one has lower proactive personality (*B* = 0.62, *p* = 0.03). Designing competition strategy was more positively related to study goal attainment when students have higher proactive personality (*B* = 1.11, *p* < 0.001), compared to when one has lower proactive personality (*B* = 0.67, *p* = 0.022). Thus, Hypothesis 3b was supported. Finally, we found a significant two-way interaction between study engagement and proactive personality on study goal attainment (*B* = 0.11, *p* = 0.03) (see Table 3, Model 6). The simple slope test showed that study engagement was more positively related to study goal attainment when students have higher proactive personality (*B* = 0.79, *p* < 0.001), compared to when one has lower proactive personality (*B* = 0.54, *p* = 0.007). Thus, Hypothesis 3c was supported.

## Discussion

Drawing on self-determination and proactivity theories (Bakker & Van Woerkom, 2017; Grant & Ashford, 2008), the current study aims to investigate whether playful study design, as a proactive behavioral strategy, can have a positive impact on study engagement and goal attainment. Using a weekly diary approach, our results generally supported our hypotheses.

First, we found the positive direct effects of playful study design, including designing fun and designing competition, on study goal attainment on a weekly basis. This implies that playful study design is indeed an effective day-to-day learning strategy to improve their engagement in study and realize study-related goals. PSD can help students enhance study engagement, fulfill study tasks, and achieve self-growth, especially during the COVID-19 period when the study environment becomes less vibrant and diverse (Maatuk et al., 2022). This finding was in line with the proactivity literature (Crant, 2000; Parker et al., 2010) indicating that proactive behaviors are one of the crucial antecedents of goal attainment because proactive behaviors are usually seen as self-mentoring and goal-oriented actions. This finding was also in line with the study of Wang et al. (2021) who reveals that weekly goal-oriented self-regulation behaviors were positively related to weekly academic performance.

We further found the mediating role of study engagement on the relationship between playful study design and goal attainment. This finding further explained why and through what mechanism playful study design has positive impact on goal attainment. We demonstrated that playful study design, including designing fun and designing competition, can activate one’s intrinsic motivation and enable individuals to reach an engaged state with dedication, vigor, and absorption, which can finally increase the likelihood of the attainment of the goal. This finding speaks to the self-determination and flow literature (Bakker & Van Woerkom, 2017; Wehmeyer, 1996) stating that self-determined strategies can lead to psychological need satisfaction, which in turn facilitates engaged/flow state. Further, we confirm that study engagement is an important positive personal state that can transform positive behaviors into favorable study outcomes (e.g., goal attainment and task completion), which echoes prior study engagement literature (see, Bakker et al., 2015; Siu et al., 2014).

Finally, we uncovered the significant moderating role of proactive personality on the relationship between PSD and goal attainment; and the relationship between study engagement and goal attainment. These results showed students with high proactive personality benefited more from using PSD and reached higher goal attainment, compared to those who scored low in proactive personality. These findings were consistent with the proactive personality literature (Porath & Bateman, 2006) indicating that proactive people are inclined to mobilize their energy and resources in the manifestation of more proactive actions. In other words, people with high proactive personality are likely to be goal-directed, so that they can keep track of their progress and create a fit between the self and personal goals (Porath & Bateman, 2006). They also tend to invest in resources and tailor their behaviors to the attainment of goals. Proactive personality is an essential personal asset that can help students to navigate and make better use of proactive strategies. However, it is noted that we did not find the significant moderating effect of proactive personality on playful study design strategies and study engagement. We also found a negative relationship between proactive personality and study engagement (*b* = − 0.35**, *b* = − 0.24** in Table 3). We explained that this might be due to a double-edged effect of proactive personality (Cangiano et al., 2020; Parker et al., 2019). On the one hand, proactive personality enables individuals to show proactivity, enthusiasm, and energy to seek challenges and pursue goals (Yi-Feng Chen et al., 2021); on the other hand, proactive personality may deplete individuals more personal resources, which can potentially increase task-related anxiety (Cangiano et al., 2019; Sprigg et al., 2007). Therefore, this may provide an explanation of why proactive personality was not positively related to study engagement and also did not moderate the relationship between playful study design strategies and study engagement. This is because students who are high in PP are open to experience, seek sensations, thus more likely being distracted by external stimuli (Parker et al., 2019). This, in turn, may make them not focus on the task at hand completely. In addition, proactive individuals may consume their energy more quickly as they are often dedicated to the task at hand (Liu et al., 2021), which may undermine their engaged states and performance after a longer period. We encourage future research to verify and examine the underlying reasons of the dark side of proactive personality (e.g., identifying important mediating mechanisms).

### Theoretical Contributions

First, we expand the playful work design studies (Bakker et al., 2020a, 2020b; Scharp et al., 2019) into the educational setting and provide students with a more proactive-oriented self-learning strategy, especially during the COVID-19 context. Prior studies have primarily investigated playful design as a proactive strategy in the work domain, and scholars have shown that playful work design was effective to improve work engagement and performance (Bakker et al., 2020a, 2020b; Scharp et al., 2019). However, playful design can also be employed in the study domain as study tasks are also complex, independent, and self-directed (Bakker et al., 2020a, 2020b). It is important to understand what students can do to deal with various study tasks and improve their performance in study tasks, because during the COVID-19 context students may feel frustrated and need to find ways to motivate themselves. Thus, our study provides one of the first attempts to apply playful design in the student learning life and explore whether it is also helpful for study engagement and goal attainment. Consequently, we extend the literature regarding proactive behavior (Crant, 2000; Parker et al., 2010; Ryan & Deci, 2000) by suggesting PSD as another effective strategy to foster study outcomes, in addition to strengths use, self-regulation, and vitality management strategies (see, Bakker & Van Woerkom, 2017). Taking together, we highlight that the playful design including adding fun and competition elements into current study tasks is beneficial and effective in both work domain and study domain.

Second, previous studies have shown that play or playful behavior is important for bringing positive outcomes, such as flow experience, intrinsic motivation, higher job performance, and life satisfaction (for a review, see Petelczyc et al., 2018). However, to the best of our knowledge, the underlying mechanism why play may profit well-being and performance remains unclear or is in lack of empirical evidence. The current study attempts to take a motivational perspective to provide insights into how playful design may help students to attain their study goals. That said, rather than focusing on learning and task performing process (Littlejohn & Margarayn, 2011; Wang et al., 2021), we underscore how one’s engaged state acts as an important intermediate anchor translating the effects of PSD on goal attainment. Thus, we add to the existing knowledge of students’ goal attainment literature (Abraham & Sheeran, 2003; Grant et al., 2009) by shedding light on how an intrinsic motivational process triggered by playful design can be an effective means to facilitate goal attainment. We also contribute to the study engagement literature by highlighting that playful study design (including fun and competition elements) is another important antecedent of study engagement in addition to study resources, study demands, and self-regulation (Bakker et al., 2015; Siu et al., 2014; Wang et al., 2021). A playful design can satisfy one’s psychological needs and activate an engagement process.

Third, we also extend proactivity literature (Crant, 2000; Parker et al., 2010) by showing how proactive behaviors (e.g., PSD) and proactive personality are important to the attainment of goals in the educational setting. Proactivity theory, in general, argues that individuals who are proactive are more likely to change themselves or environments, obtain social resources, have better performance, and achieve career success (Elsaied, 2019; Grant & Ashford, 2008; Parker et al., 2019). Our findings echo this stream of argumentation or research by showing that proactive personality moderates the effects of PSD on goal attainment and the effects of engagement on goal attainment. This implies that proactive personality is an important personal asset to safeguard the implementation of proactive behaviors. Examining the moderating role of proactive personality, we shed light on what personal traits/abilities can maximize the benefits of PSD strategy and engage more in the proactive study process. In sum, this study uncovers not only why and how PSD can lead to beneficial study outcomes, but also from whom PSD can unleash more potential.

Fourth, to advance the understanding of the relationships among PSD, study engagement, and study goal attainment, we employed a weekly diary approach. That said, we did not simply uncover how those engaging in PSD (vs. those not) relate to higher study engagement, and in turn, facilitate goal attainment (i.e., between-person variations). More importantly, we present how PSD influences study engagement and goal attainment within the short period of time (i.e., within-person variations). Thus, we add to the study engagement and performance literature (Bakker et al., 2015; Siu et al., 2014; Wang et al., 2021) by highlighting the importance of examining the dynamic fluctuations of students’ behaviors and states. We provide more insights into how PSD acts as an ongoing action influencing the changes of student engagement and goal attainment over time.

Finally, we highlight an important context—the COVID-19 period—and how playful study design can help students fit such an unfavorable study context. The research has shown that the COVID-19 pandemic has brought more challenges for universities and students (Maatuk et al., 2022). For example, studies showed that the online learning format due to the lockdown of COVID-19 was negatively related to skill mastery, social competences, and being active (Bączek et al., 2021). Some studies realized the importance of changing teachers’ teaching strategies, such as providing brief, clear, and interesting learning materials; choosing a simple and attractive learning media (Sutarto et al., 2020). However, less is known about what students themselves can do to adapt to an online learning environment and keep their study motivation. Thus, our study suggests a useful means for students’ self-learning during COVID-19 period. That is, we highlight that adding fun and competition elements into the study process can effectively activate study engagement state, and in turn, reach a higher level of goal attainment. Thus, we provide valuable insights into how PSD can be considered as an effective learning strategy under an unfavorable study condition. We recognize PSD as an effective study tool for students to attain their study goals and to make their study fun and meaningful when they experience difficulties in a face-to-face class, teacher–student/student–student interactions, and a turbulent study environment.

### Practical Implications

Our study provides valuable practical implications for both students and educators. Since the current study empirically showed that students will be more engaged when they use PSD, we encourage students to leverage this self-determination strategy more often in their learning life. For example, when students feel tasks boring, they can proactively improve task challenges to make the tasks more competitive. If a student has a good sense of humor, they can use this strategy when conversing with others, such as experiencing more enjoyment during the conversation. Moreover, for students who have low PP, as the PSD does not function as effectively as it is for those who have high PP, they may need more support for using PSD.

From a top-down perspective, an organization (e.g., school, university) can teach and train their students to use this self-determined strategy. For example, headmasters, educators, and teachers can nudge students and give them autonomy to find more fun and joy within their study tasks. It will be not complicated to do so as tiny changes in task challenges probably allow students to be more immersed in task at hand. Besides, university professionals or practitioners can develop relevant training programs to stimulate and cultivate students to engage in self-determination study strategies (e.g., PSD). Teachers can also try to establish a balance between study task challenge and task fun when they assign study tasks. By doing so, students would not feel the tasks too boring or too difficult, which can, in turn, enhance their study engagement.

### Limitations and Future Research

The current study is not without limitations. First, all the measures are based on self-reports, which may potentially increase the likelihood of common method bias (Podsakoff et al., 2003). However, as we have conducted multilevel factor analysis, the measured constructs indeed can be distinguished from each other, demonstrating that our measurements are validated. Moreover, as we used a weekly diary approach, and conducted person-mean centering, which enabled us to focus on within-person fluctuations rather than associations at a between-person level. This analyzing approach also mitigates the risks of common method bias (Scharp et al., 2021). Future studies are encouraged to use more objective measures reading PSD, engagement, and goal attainment, such as other-rated goal attainment. It is also noted that we only recruited participants from one business school. Although we tried other schools to improve the generalizability of our study but only one school actively responded. Thus, we encourage future studies to further replicate our findings in other school settings.

Second, the current study cannot establish any causal relationship between the study constructs. Even though it is theoretically indicated that PSD increases study engagement, which in turn, improves goal attainment, our findings are drawn from associated (or relational) relationships rather than causal associations. However, as we have controlled for the lagged effects, which means the engagement and goal attainment level in the previous week, we obtain more robust and causal-like results (Hamaker & Grasman, 2015). This autoregressive analysis approach has the advantage the look into the fluctuating relationships more stringently regardless of the initial level of the dependent variable (Liu et al., 2021).

Third, future studies should also investigate how contextual and environmental factors act on PSD. Except for key personal resources (e.g., emotional intelligence, personality), external demands and contextual resources may also play a role in determining the effects of PSD on engagement (Bakker & Van Woerkom, 2017). As the inclusion of more environmental variables is beyond the scope of the current study, we encourage future studies to investigate the external stakeholders that may play a role in student study life. For example, will teachers’ strategy or style interact with PSD to together determine student motivation and study engagement? Is it the case that students who receive more social support likely flourish using PSD? Future studies can explore the association between these external factors and PSD.

## Conclusion

The current study demonstrates that playful study design can be used as an effective strategy for students to improve engagement and goal attainment at study. We particularly highlight that PSD is an effective strategy for students and education managers during the period of COVID-19 when the study circumstances become unfavorable and uncertain. Students can use this strategy to self-manage their study motivation process and achieve various study goals. Education managers can promote PSD as well by encouraging more students to apply this self-motivating strategy.

## Data Availability

The data can be obtained via request from the corresponding author.
